# Mortality Pattern in Children: A Hospital Based Study in Nigeria

**Published:** 2009-12

**Authors:** I. O. George, B. A. Alex-Hart, A. I. Frank-Briggs

**Affiliations:** *Department of Paediatrics, University of Port-Harcourt Teaching Hospital, Port-Harcourt, Nigeria*

**Keywords:** mortality pattern, children, admission, HIV/AIDS, MDGs

## Abstract

Background: Hospital based data on mortality pattern is a reflection of what is obtainable in a community at large. Therefore, data obtained from such review is usually beneficial in re-evaluating existing services and in improving facilities and patient care. The aim of this study was to evaluate the mortality pattern of children admitted into the children medical wards of the University of Port-Harcourt Teaching Hospital from Jan 2007 to December 2008. Materials and Methods: This was a retrospective study. The case files of all patients aged one month to 16 years, admitted into the paediatric wards of University of Port-Harcourt Teaching Hospital, Port-Harcourt, Nigeria over a 2 year period were reviewed. Neonatal and surgical cases were excluded. Results: There were 2,174 admissions during the study period. Sixty one of the total number of admissions died in the children medical wards giving a mortality rate of 2.8%. The youngest child was 2 months and the oldest 10 years. Fifty two (80.3%) were under 5 years. There was male preponderance. Most of the deaths occurred between April and September. The commonest causes of death were HIV/AIDS and bronchopneumonia in the under five age group; while in those above 5 years of age malignancies and HIV/AIDS were the predominant causes. Conclusion: Effective HIV/AIDS control measures will significantly reduce child mortality in our community. Also there is need to have a closer look at the potential risk for malignancies. Health intervention programmes such as integrated management of childhood illnesses and primary health care, which have been shown to reduce childhood deaths significantly, need to be intensified in order to achieve the MDG 4 by 2015.

## INTRODUCTION

Child mortality is a sensitive indicator of a country’s development and telling evidence of its priorities and values (1). Every day, more than 26,000 children under the age of five die around the world, mostly from preventable causes (2). Nearly all of them live in the developing countries (2). More than one third of these children die during the first month of life, usually at home and without access to essential health services and basic commodities that might save their lives. Some children succumb to respiratory or diarrhoeal infections that are no longer threats in industrialized countries or to early childhood diseases that are easily prevented through vaccines, such as measles. In up to half of under-five deaths an underlying cause is under nutrition, which deprives a young child’s body and mind of the nutrients needed for growth and development (3). Unsafe drinking water, poor sanitation and inadequate hygiene also contribute immensely to child mortality and morbidity (3).

Childhood deaths have been reported to be concentrated in poor resource settings like Nigeria where poverty, ignorance and social instability have provided a platform on which malnutrition and infection-related diseases have resulted in childhood deaths (4). Impressive progress has been made in improving the survival rates and health of children, even in some of the poorest countries, since 1990 (5). However it is worrisome to note that high rate of infant and child morbidity and mortality is still one of the greatest challenges facing most of the countries in Sub-Saharan Africa (1). This study is therefore aimed at evaluating the mortality pattern in children in the University of Port-Harcourt Teaching Hospital (UPTH). The teaching hospital is located in the metropolis of Port Harcourt, the capital of Rivers State, southern Nigeria. The hospital serves as a general/referral centre for neighboring states. The information obtained from this study would be used in re-evaluating existing services and in improving facilities and patient care.

## MATERIALS AND METHODS

This was a retrospective cross sectional descriptive study over a two year period from January 1, 2007 to December 31, 2008. The case files of all children aged one month to 16 years admitted into the paediatric wards as recorded in the ward register, were reviewed. Neonatal and surgical cases were excluded from the study. Data extracted from the case files included age, gender, principal diagnosis, cause of death, and duration of hospitalization. The principal diagnosis was based on the final assessment by the managing unit. It was based on the presenting clinical features, with or without the results of laboratory tests. For instance, the diagnosis of malaria was supported by the presence of malaria parasites in the blood film. Patients with bronchopneumonia were diagnosed based either clinically or by chest radiographs or both. HIV/AIDS was based on positive Elisa test on a patient with features of the WHO clinical case definition of HIV/AIDS in Africa, which was confirmed by Western blot test. Diagnosis of meningitis was based on the clinical features with or without positive culture or abnormal biochemical analysis while that of malignancies were based on clinical features, ultrasound report and biopsy results. The cause of death as documented after weekly mortality reviews was considered as the final cause of death.

Data collected was entered into a spread sheet using SPSS 15.0 for Windows^®^ statistical software which was also used for analysis. Descriptive statistics was used to analyse the obtained data.

## RESULTS

There were a total of 2,174 admissions into the paediatric wards during the study period. Sixty one deaths were recorded. These were made up of 34 males and 27 females giving a ratio of 1.3:1. Fifty two (85.2%) were under the age of five years with peak age between 2 months to 2 years (Table 1). The cumulative monthly mortality shows that there were more deaths between April and September (Table 2). Table 3 shows the main causes of death among children who are under five years. HIV/AIDS was the commonest cause of death in 11 (21.2%), followed by bronchopneumonia in 8 (15.8%). All the HIV patients had features of full blown AIDS and their ages ranged from 9 months to 6 years. The main causes of death in those above 5 years are as shown in Table 4. Malignancies and HIV/AIDS were the commonest cause of death (33.3%) respectively. Those who died of malignancy had neuroblastoma and nephroblastoma.

**Table 1:** Age and sex distribution in 61 mortalities

Age interval	Number of deaths	
	Male	Female

2 months – 2 years	23	22
>2 – <5 years	6	1
>5–10 years	5	4
Total	34	27

**Table 2:** Cumulative quarterly mortality

Months	Number of deaths	Percentage

January-March	10	16.4
April-June	21^a^	34.4
July-September	19^a^	31.2
October-December	11	18.0

a Common causes of death from April-September: HIV (n=10), pneumonia (n=5), malaria (n=4), diarrhoea (n=3).

**Table 3:** Causes of death in the under five children

Conditions	Number of patients	Percentage

HIV/AIDS	11	21.2
Bronchopneumonia	8	15.4
Chromosomal abnormalities with cardiac defect	7	13.5
Renal diseases	6	11.5
Severe malaria	5	9.6
Malignancies	5	9.6
Tuberculosis	4	7.7
Diarrhoea diseases	3	5.8
Meningitis	2	3.8
Congenital heart diseases	1	1.9
Toal	52	100

**Table 4:** Causes of death in the above five years

Causes	Number of patients	Percentage

Malignancy	3	33.3
HIV/AIDS	3	33.3
Tetanus	2	22.2
Sickle cell disease	1	11.1

## DISCUSSIONS

The overall mortality of 2.8% in this study was similar to previous studies in our centre (6) but lower than the 6.8 % observed in Lagos, western Nigeria (7). This reason for this lower mortality might be as a result of methodology adopted in this study which excluded neonatal deaths and deaths from children emergency ward. It is possible that prompt and appropriate health seeking behaviour by the parents accounted for the observed differences.

The seasonal variation in mortality had been described all over the world (8). We observed that most of the deaths occurred between the months of April and September. This period coincides with the wet or raining season. Increased mortality during the wet season may be explained by our finding that the major causes of deaths such as bronchopneumonia, malaria and diarrhoea occur more frequently during the wet season. This is because the wet season encourages breeding of mosquitoes and provides chilling environment for micro-organisms to cause pneumonia and contaminates our sources of drinking water. This pattern had been reported by previous Nigerian studies (7, 9).

This study has shown that over 80% of deaths that occurred were in children under the age of five. This reflects the vulnerability of this age group. However, HIV/AIDS and bronchopneumonia were the main cause of death in this series resulting in over 30% of the mortality in this age group. This is in conformity with previous Nigerian (9) as well as other African studies (10, 11). HIV/AIDS alone accounted for 21.1 % of under five deaths. This figure is comparable to a study done by Ojukwu and colleagues at Abakiliki, eastern Nigeria where mortality rate of 14.3 percent was recorded (9). It is worthy of note that deaths from diarrhoea diseases were lower than earlier studies (9, 12). This is as a result of increased awareness and use of oral rehydration solution by most mothers, more appropriate feeding practices, improved sanitation and the use of oral zinc supplementation (13, 14).

Malaria is a leading cause of mortality in children under the age of 5 years in Nigeria (15). In our study deaths due to malaria is high (9.6%) compared to studies from other centers in Nigeria (16, 17), where malaria related deaths were low among the under fives. This may be a result of high levels of parasite resistance to affordable drugs, and late presentation for treatment at the hospital. Implementation of the goals of the roll back malaria in Nigeria will save many children from dying from malaria.

Tuberculosis (TB) is an important cause of mortality in this series where it accounted for 7.7% of the mortality rate in children under 5-years of age. It is possible that the HIV/AIDS pandemia and delay in presentation were responsible for these deaths. In sub-Saharan Africa, TB is felt to be the most commonly diagnosed opportunistic infection, and it is also the most frequent cause of death among those infected with HIV (18). Infection with HIV increases the likelihood of progression to active tuberculosis which changes the clinical manifestation of the disease. The immune stimulation caused by TB may also increase the HIV viral load, rate of HIV disease progression, and mortality, particularly among those with higher CD4 counts (18). Delays, however, may occur as a result of difficult access to the hospital, seeking treatment in the traditional or private sector and delay in making diagnosis of TB. These may compromise the chance of a successful outcome, and lead to increase transmission of TB both in the household and in the community.

The emergence of non-infectious disorders such as malignancies as the main cause of mortality in those children above 5 years of age is worrisome. Nephroblastoma (n=2) and neuroblastoma (n=1) were the only malignant condition that lead to the death of these children in this study. However, this pattern is similar to that reported in other centers (16, 17) in Nigeria where mortality rates were 23.8% and 4.8% respectively. This calls for intensified researches into identifying early symptoms, risk factors and means of prevention in order to reduce morbidity and mortality due to childhood cancers. It is possible that those children who died from malignancy might be due to the disease itself or related to treatment. Treatment for malignancy can deplete normal host defences, change local anatomy and disrupt natural barriers to infection resulting in a major predisposition to infection. It is therefore not surprising that deaths due to malignancies are the leading causes of mortality in children particularly in this region where infections have contributed greatly to childhood mortality (16, 17).

In conclusion, this study has shown that childhood mortality remains high in children under the age of 5 years and is mainly due to infectious and other preventable causes such as HIV/AIDS, bronchopneumonia and malaria. Infant and child mortality remain disturbingly high in developing countries, like Nigeria, despite the significant decline in most parts of the developed world (19). In view of the fact that the major childhood diseases have been identified and modern technology to combat them developed, yet, children from African countries (Nigeria inclusive) die in large number from the attacks of these diseases. The adduced reason is deeply rooted in people’s beliefs and attitudes concerning childcare and behavioural practices into health strategies. For instance, measles attack is traditionally considered as a punishment for breaking family taboos or as an evil deed from witches or enemies (19). Also, it is believed that diarrhoea is caused by excessive consumption of sweet things and for this, mothers would not accept use of oral rehydration solution for treatment since it contains sugar (19). Nigerian health policy recognizes the need to reduce the current high childhood mortality, the people’s belief and behavioural practices have not been adequately integrated into health intervention programmes. It is disturbing to find out that people are still holding on to their wrong perceptions and attitude towards the etiology of certain childhood diseases and deaths despite the positive effect that modernization and education are having on people’s behaviour. Many people have not realized that infant and child mortality result from the combined effects of nutritional deficiencies, infections, parasitic and respiratory diseases. Therefore, there is need to integrate the people’s beliefs, attitudes and behavioural practices into health promotion programmes to achieve a maximum reduction in child and infant morbidity and mortality. Unless this is done, there might not be too much progress as regards reduction of infant and childhood morbidity and mortality in Nigeria. Also, health intervention programmes such as integrated management of childhood illnesses (IMCI) (20) and primary health care (21) which have been shown to reduce childhood deaths significantly, need to be intensified in order to achieve the MDG 4 by 2015.
